# Botanical Description of British Plants

**Published:** 1808-01

**Authors:** 


### *Botanical Description of British Plants.*

[ Continued from Vol. xviii, pp. 457—461. ]

### Class XVII. Diadelphia.

### Diadelphia. Hexandria.

1. Fumaria. *F. officinalis.—F. purpurea. F. vulgaris.*

*Ang.* Fumitory.

*Gen. Desc.* Cal. two-leaved. Bloss. gaping, bulging at the base, containing honey. Filaments two, membranaceous, each supporting three anthers. Caps. one celled, many seeded.

*Spec. Desc.* *Seed-vessels* in bunches, one-seeded, roundish, smooth. *Stem* spreading, smooth. *Leaves* smooth, somewhat fleshy, sea green, trebly compound, the last div. with five or three clefts; extreme segm. spear-shaped. *Floral-l.* spear-shaped, membranaceous, one at the base of each fruit-st. *Fruit-st.* v. short. *Cal.* coloured. *Bloss.* alternate, in long terminating spikes, pink and dark purple, sometimes pale; upper lip keeled at the back, reflected towards the end. *Nect.* distended, inclosing a greenish tongue-shaped substance, purple at the edge.—*Corn-fields, hedge banks.* Bl. May, Aug.

*Use.* The leaves, which are the part directed for medicinal use, are extremely succulent, have no remarkable smell, but a bitter somewhat saline taste. The expressed juice, or a decoction of the leaves in water, inspissated to the consistence of extracts, are very bitter and considerably

---

* Vide, Medical Museum, Vol. I, page 101.

bly saline; or standing for some time they throw up copious saline efflorescences, in figure resembling the chrystals of nitre, bitterish and slightly pungent to the taste.—*Lewis.* Fumitory has been considered by physicians of great authority to be very efficacious in opening obstructions and infarctions of the viscera, particularly of the hepatic system: it has also been employed in various cutaneous diseases as a corrector of a scorbutic and acrimonious state of the fluids: taken in large doses it is diuretic and laxative, especially the juice, which may be mixed with whey and used as common drink. Dr. Cullen classes this plant among the tonics, and says that its remarkable virtues are those of clearing the skin of many disorders; its good effects in this way he experienced in many instances of cutaneous affections: he used the expressed juice, giving to 2 oz. twice a day; but the virtues remain in the dried plant, so that they may be extracted by decoction or infusion of it in water. *(Cullen ii. 77.)* The juice of fumitory and dandelion is greatly commended by Leidenfrost in obstinate diseases of the skin. This plant was thought by the ancients peculiarly useful in dimness of sight, and other disorders of the eyes.—*Woodville.*

The expressed juice in doses of 2 or 3 oz. is useful in hypochondriacal, scorbutic, and cachectic habits. It corrects acidity and strengthens the tone of the stomach. Hoffman prefers it to all other medicines as a sweetener of the blood. There is no doubt of its utility in obstructions of the viscera and diseases thence arising. An infusion of the leaves is used as a cosmetic to remove freckles and clear the skin.—Cows and sheep eat it; goats are not fond of it; horses and swine refuse it.—*Withering.*

Diadelphia. Octandria.

2. Polygala. *P. vulgaris.*

*Ang.* Milk wort.

*Gen. Desc.* Cal. five-leaved; two larger segm. wing-like, before the seeds ripen, coloured. Caps. inversely heart-shaped, two celled; seeds solitary.

*Spec. Desc.* *Stem* herbaceous, simple, trailing, four cornered, undivided. *Leaves* strap-spear-shaped, a little rolled back. *Flowers* in bunches, with a pencil-shaped appendage, blue, purple, flesh colour, or white; *wings* spear-shaped; *standard* 2 petals joined by the hairy edges; *keel* cylind. below, expanding upwards into two sets of club-shaped, glandular appendages. *Filam.* two sets, like two hands, four fingers to each. *Anthers* yellow or orange.

orange. *Bunches* terminating.—*Pastures and heaths.* Bl. June, July.

*Use.* It was found by Linnæus to possess the same virtues as the Senega rattle-snake, Polygala Senega, which is imported from America, but in an inferior degree. It purges without danger; is emetic and diuretic, and sometimes acts in these three different ways together. A spoonful of the decoction (1 oz. of the herb boiled in one pint of water till half exhaled) has been found useful in pleurisies and fevers, by promoting a diaphoresis and expectoration: three spoonfuls of the same taken every hour has proved useful in dropsy and anasarca: it has also been found beneficial in phthisicky complaints.—*Lightfoot.* Duhamel used it in pleuritic cases with the desired success. *Mem. de Paris*, 1740. The powdered root may be given in doses of half a drachm. An infusion of the herb, which is very bitter, taken in the morning fasting, about a quarter of a pint daily, promotes expectoration, and is good for a catarrhous cough; Dr. Smith reports that he tried it with success.—Cows, goats, and sheep eat it; swine refuse it.—*Withering.*

Diadelphia. Decandria.

3. Spartium. *S. scoparium.*—*Genista angulosa et scoparia.*

*Ang.* Common broom.

*Gen. Desc.* Cal. extending downwards, two lipped. Filaments adhering to the germen. Stamens all united. Summit woolly above, growing on the side of the style.

*Spec. Desc.* *Leaves* in threes, and solitary, slightly hairy. *Legumen* fringed with soft hairs. *Branches* without prickles, angular. *Cal. upper segm.* two tooth: *lower* three toothed. *Bloss.* yellow; standard nearly circular, slightly notched; *keel* petals hooked, united at the lower edge by an intertexture of soft hairs. *Stam.* four long, six short. *Style* bowed into a circle, after flowering into a spiral, the very end not hairy.—*Dry pastures.* Bl. May, June.

*Use.* The tops and leaves have a nauseous bitter taste: they are commended for their purgative and diuretic qualities, and have therefore been successfully employed in hydropic cases, of which particular instances are related by Dr. Mead, and others: a patient by drinking half a pint of a decoction of the green tops, with a spoonful of whole mustard seed, every morning and evening, was cured, after being tapped three times, and having tried the usual remedies remedies given in dropsies. *Mead monita et præcep. Med. p.* 138. An infusion of seeds drank freely has been known to produce the same happy effects; but Dr. Withering remarks, that whoever expects these effects to follow in every dropsical case will be greatly deceived; he knew them succeed in one case that was truly deplorable, but out of a great number of fair trials, he adds, that was a single instance.—*Withering.* A strong lixivium of the ashes was used in the Swedish army, in 1759, to cure dropsies consequential to catarrhal epidemic fever; the urine plentiful and the dropsies soon disappeared. *Med. comm. i, p.* 373. Dr. Cullen says, that though very little in use, he has, on his own experience of it, inserted broom in his catalogue of cathartics; he prescribed it thus: half an ounce of fresh tops was boiled in 1lb of water till one half of this is consumed; of this decoction he gave two table spoonfuls every hour till it operated by stool, or till the whole was taken; it seldom fails, he adds, to operate both by stool and urine, and by repeating this exhibition every day or every second day, some dropsies have been cured. *M. M. ii.* 534. The ashes have been much used in dropsies on the authority of Sydenham, whose account of their good effects has been since confirmed by Dr. Monro, (he gave a drachm divided into three doses every day. *Monro, on Dropsy, p.* 64) and by other writers. The efficacy of this medicine depends entirely upon the alkaline salt, and not at all upon the vegetable from which it is obtained.

The seeds and flowers are said to be emetic and cathartic, but the evidence of this is not to be relied on, as the former roasted have been used as a substitute for coffee, and the latter employed as a pickle. Ray asserts, on the authority of a MS. of Dr. Hulse, that the flowers given in the form of an electuary with honey of roses, were, in scrophulous affections, found very efficacious.—*Woodville.* The flowers and seeds from 2 drs. to ½ an oz. are a strong vomit. The tender branches are not inferior to oak bark for tanning; and they are sometimes mixed with hops for brewing. The bark macerated may be manufactured into cloth. The old wood is valuable for vaneering.—*Lightfoot.* The young flowers are sometimes used as pickles; and the seeds are by some people toasted, so as to be used in the manner of coffee. This plant when burnt affords a tolerably pure alkaline salt. When growing large the broom merits a place among flowering shrubs, on account of its large golden coloured blossoms.—Cows, horses, and sheep refuse it.—*Withering.*

4. GENISTA.

4. Genista. *G. tinctoria.*

*Ang.* Dyer's green wood. Green wood. Dyer's weed. Wood waken.

*Gen. Desc.* Cal. two-lipped; up. two-toothed; lower three-toothed. Standard oblong, bent back from the pistils and stamens. Pistil pressing down the keel. Legumen regular shaped.

*Spec. Desc. Branches* scored, cylind. upright. *Leaves* spear-shaped, smooth. *Legu.* cylind. *Flowers* in leafy spikes, yellow. *Stand.* egg-shaped; *wings* oblong-oval; *keel* compressed; *Summ.* a knob. *Cal.* five-cleft.—*Pastures, borders of corn-fields.* Bl. July, Aug.

*Use.* A drachm and a half of the powdered seeds operates as a mild purgative; a decoction of the plant is sometimes diuretic, and has therefore been found serviceable in dropsical cases. A yellow colour may be prepared from the flowers; and for wool, which is to be dyed green, it is preferred by the dyers to all others.—Horses, cows, sheep, and goats eat it.—*Withering.*

5. Ulex. *U. europæus.*

*Ang.* Furze. Gorze. Whins.

*Gen. Desc.* Cal. two-leaved. Legumen scarcely longer than calyx.

*Spec. Desc. Leaves* woolly, acute; thorns scattered. *Flowers* bright yellow, sometimes though rarely white, at the base of the thorns.—*Heaths, road sides, pastures.* Bl. Feb. Aug.

*For Var. see Bot. Arr. This blossoms all the year, but most fully in Sept. and Oct.*

*Use.* In Cornwall where fuel is scarce, and in many parts of Ireland, it is cultivated to advantage, and is generally cut to make faggots for heating ovens, which it does very soon, burning rapidly, and with a great degree of heat; in some places in the vicinity of Cork, the land is said to yield a larger profit under this plant than it would bring under tillage. The ashes are used to make ley. In some respects it is a very hardy plant, and will make fences upon the bleakest mountains, and close to the sea, where the spray destroys almost all other shrubs; but it is impatient of cold, and is often destroyed by severe frosts; it is rarely found in the Northern part of the Island. Team horses may be supported by this plant, if it be cut young and bruised in a mill to break the thorns.—Horses, cows, sheep, and goats are fond of feeding upon the tender tops.

6. Ononis. *O. spinosa.*

*Ang.*

*Ang.* Thorny rest-harrow. Cammock. Petty whin. Ground furze.

*Gen. Desc.* Cal. five-div. Segm. strap-shaped. Standard scored. Filaments united without an opening. Legumen swoln, diamond-shaped, sitting, simple, one-celled.

*Spec. Desc.* *Leaves* solitary, or in threes, nearly smooth. *Branches* thorny. *Flowers* in bunches, mostly solitary. *Bloss.* twice as long as the cal. red. *Cal.* hairy, segm. awl-shaped, unequal. *Thorns* awl-shaped, with sometimes one or two leaves. *Stems* prostrate, woolly, reddish, thorny, wooddy.—*Barren pastures, hedge banks, hollow ways.* Bl. July.

*Use.* The root and bark have a diuretic quality, and are recommended in the gravel, and in suppressions of urine, both for man and beast.—*Lightfoot.* A decoction of the root has been recommended in cases of stone and jaundice. Cows, horses, and goats eat the young shoots; sheep are very fond of it; swine refuse it.—*Withering.*

7. Anthyllis. *A vulneraria.*

*Ang.* Kidney vetch. Lady's finger.

*Gen. Desc.* Cal. bellying. Legumen roundish, covered.

*Spec. Desc.* *Stem* herbaceous, cylind. downy. *Leaves* winged, unequal, downy. *Flowers* in a double head, sitting, deep yellow in England, (scarlet in Portugal. *Filam.* of a singular structure. *See Bot. Arr.* *Meadows, in chalk or calcareous soil.*—Bl. May, Aug.

*Use.* It is used by the country people as a yellow dye. It makes excellent pasturage for sheep. It is observed by Linnæus, that when growing in reddish clay its blossoms were red, but when in white clay they were white.—Cows and gots eat it.—*Withering.*

8. Pisum. *P. maritimum.*

*Ang.* Sea pea.

*Gen. Desc.* Cal. 2, upper segments shorter; style with 3 angles, keeled and pubescent above. Legumens inflated.

*Spec. Desc.* Plant downy. *Stem* angular, trailing, short. *Leaf-stalks* flattish above, with many leafits, with terminating tendrils. *Stipulæ* arrow shaped. *Fruit-st.* many flowered. *Bloss.* towards the end of the fruit-st. crowded, on short pedicles, pale red and purple. *Leaves* numerous, alternate. *Leaf-scales* in pairs, opposite. *Sea-shores* Bl. Aug.

*Use.* In the year 1555, during a time of great scarcity, the people about Orford in Sussex, were preserved from perishing, by having recourse for food to the seeds of this plant, which grew in great abundance on the neighbouring seasea-coast.—Horses, cows, sheep, and goats eat it.—*Withering.*

9. OROBUS. *O. tuberosus.*

*Ang.* Heath peaseling.

*Gen. Desc.* Cal. blunt at the base; 2 upper teeth shorter, but more deeply divided; style thread shaped.

*Spec Desc.* *Leaves* winged, spear-shaped; leafits 1 to 3 pair, sitting, eliptical; *upper* narrower, nearly strap-shaped, no odd one; leaf-stalk extended into a sort of point. *Stipulæ* half arrow-shaped, entire, in pairs at the base of the leafstalks. *Stem* simple, at first drooping, when in flower upright, with 2 or 3 membranaceous leafy edges. *Bloss.* purple, 3 or 4 together. *Seeds* compressed about 12. *Legum.* flat, black, pendant. *Moist heaths and woody meadows*—BL. April, May.

*Use.* The roots, when boiled, are savoury and nutritious; ground to powder they may be made into bread. They are held in high esteem by the Highlanders of Scotland, who chew them, as the English do tobacco, and find that they prevent the uneasy sensation of hunger; they imagine them to be very efficacious in curing disorders of the lungs, and think that they promote expectoration. *Pennant* 1772, p. 310. *Ray. Hist.* The Highlanders chew the tubercles of the roots to give a better relish to their liquor; they are said to be useful in most disorders of the thorax, and the use of them is able to repel hunger and thirst for a long time. Bruised in water they make an agreeable fermented liquor; they taste like liquorice, and, when boiled, are well flavoured, and nutritive; and in times of scarcity they have been used as a substitute for bread.—Horses, cows, goats, and sheep eat it.—*Lightfoot.*

10. LATHYRUS.

The seeds of every species of this genus are nutritious, either eaten in broth, or made into bread, viz.

1. L. appaca, *yellow vetchling.*
2. L. nissolia, *crimson grass vetch, or vetchling.*
3. L. sylvestris, *narrow-leaved pease, everlasting, or vetchling.*
4. L. hisrutus, *rough-podded vetchling.*
5. L. pratensis, *tare everlasting, common yellow, or meadow vetchling.*
6. L. latifolius, *broad-leaved pease, everlasting, or vetchling.*
7. L. palustris, *chickling vetch, marsh vetchling.*

*Gen. Desc.* Cal. 2, upper segments shorter, style flat, broader broader upwards, woolly in the upper surface. Legumen generally equal, broad. Filam. 9, united, 1 distinct.

11. Vicia. *V. Sativa.*

*Ang.* Common vetch. Fitch. Tare.

*Gen. Desc.* Summit bearded across underneath. Segm. with knot-like protuberances.

*Spec. Desc.* Legumens mostly in pairs, upright. *Leaves* winged; *leafits* about 6 pair, opposite, blunt, inversely spear-shaped, notched, mid-rib lengthened to a projecting point. *Stipulæ* toothed, in pairs, spear-shaped, marked with a black spot underneath. *Stem* upright, scored. *Flowers* mostly by twos, sometimes solitary, reddish purple. *Seeds* 10 to 12, black, compressed. *Dry meadows, corn fields.* Bl. April, June.

*Use.* A decoction of the seeds in water is sometimes given by nurses to expel the small-pox and measles. In some parts of Sweden they are used for bread, either alone or mixed with rye. It is excellent fodder for horses, and a crop of it ploughed in is a very good manure, *Lightfoot.* In Gloucestershire and Worcestershire this is sown as pasturage for horses, and eaten off early enough to allow of turnips being sown the same year. The seeds are excellent food for pigeons.—Horses, cows, sheep and goats eat it.—*Withering.*

12. Trifolium.

Of the flowers of all the species of this genus, dried and powdered, bread may be made, which in seasons of scarcity has preserved the inhabitants of Scotland from perishing. The leaves of all the species fold up before rain.—*See With. Bot. Arr. l. c.*

( To be continued. )

